# Features of ductal carcinoma in situ ultrasound images

**DOI:** 10.1007/s10396-023-01334-7

**Published:** 2023-06-28

**Authors:** Takanori Watanabe

**Affiliations:** National Hospital Organization Sendai Medical Center, 2-11-12 Miyaginohara, Miyagino-ku, Sendai, Miyagi 983-8520 Japan

**Keywords:** Breast cancer, Ultrasound, DCIS, Non-mass lesions

## Abstract

Ultrasound images of ductal carcinoma in situ (DCIS) show a wide range of variations from mass to non-mass lesions. This article describes the characteristics of ultrasound images of DCIS based on the BC-02 study conducted by The Japanese Association of Breast and Thyroid Sonology (JABTS). In the BC-02 study, ultrasound images of 705 DCIS cases were classified by imaging findings. The results showed that non-mass abnormalities accounted for 60% of all lesions and masses for 40%. Looking at each subclassification, hypoechoic areas in the mammary gland were the most common (50% of the total), followed by solid masses (31%), mixed masses (9%), and abnormalities of the ducts (8%). These four classifications accounted for 98% of the total. Echogenic foci without a hypoechoic area, architectural distortion, and clustered microcysts were very rare, accounting for about 1% of the total. The ultrasound images of DCIS were characterized by a wide range of variations from masses to non-masses abnormalities, with hypoechoic areas in the mammary gland being the most common, followed by solid masses.

## Introduction

Ductal carcinoma in situ (DCIS) accounts for about 10% of all breast cancers. The Japanese Association of Breast and Thyroid Sonology (JABTS) introduced the concept of non-mass abnormalities in 2004 and classified DCIS ultrasound images [1]. The topic of this paper is the characteristics of ultrasound images of DCIS. Ultrasound images of invasive cancer are primarily recognized as masses. However, ultrasound images of DCIS are characterized by a variety of sonographic features, ranging from masses to non-mass abnormalities that are not recognized as masses. Since DCIS presents a variety of ultrasound images, it is important to understand the variations. This article describes the ultrasound imaging characteristics of DCIS based on the results of the JABTS BC-02 study [2], which reviewed 705 DCIS ultrasound images, conducted by the Japanese Association of Breast and Thyroid Sonology (JABTS).

## Classification of DCIS using ultrasound imaging

**Fig. 1:**
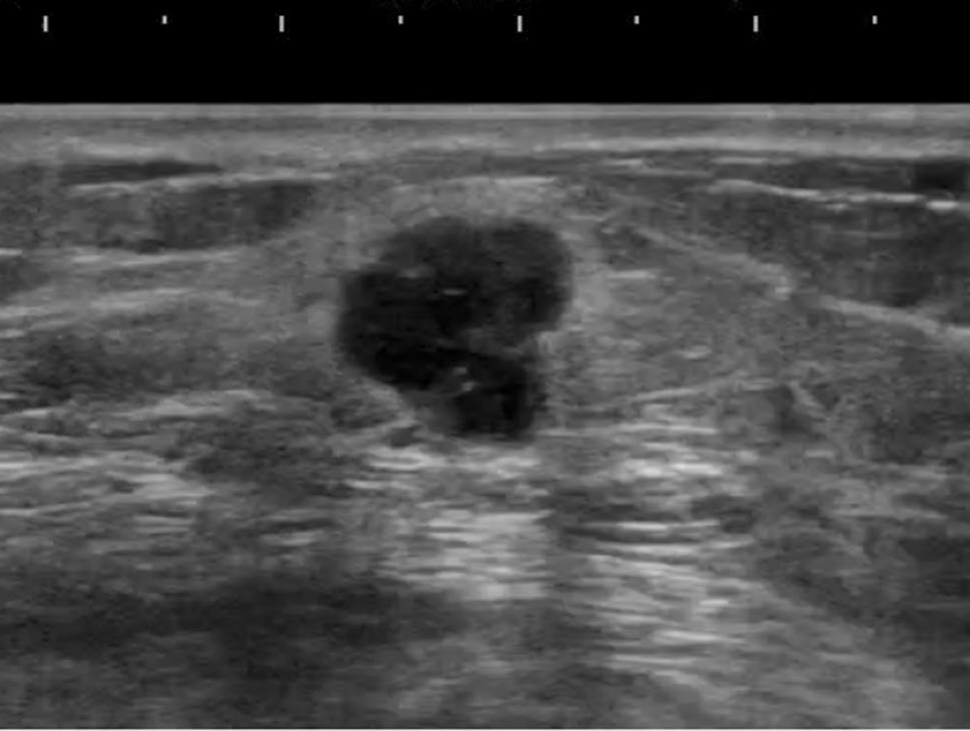
Typical image of solid masses

**Fig. 2:**
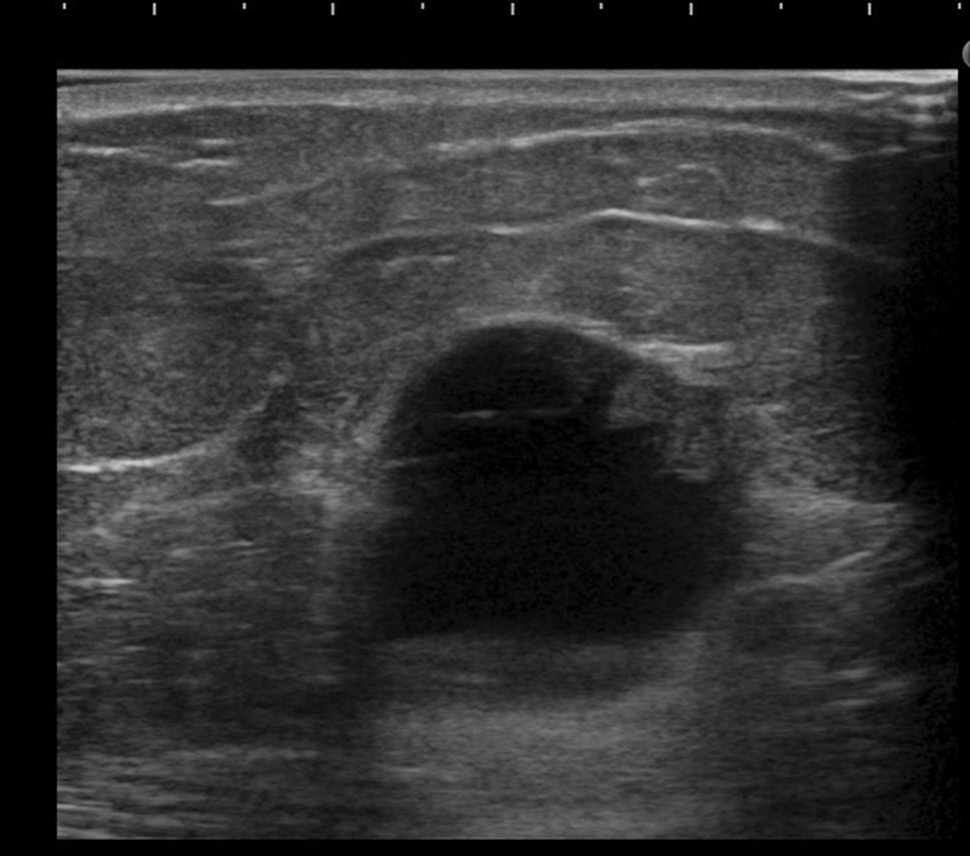
Typical image of mixed masses

**Fig. 3:**
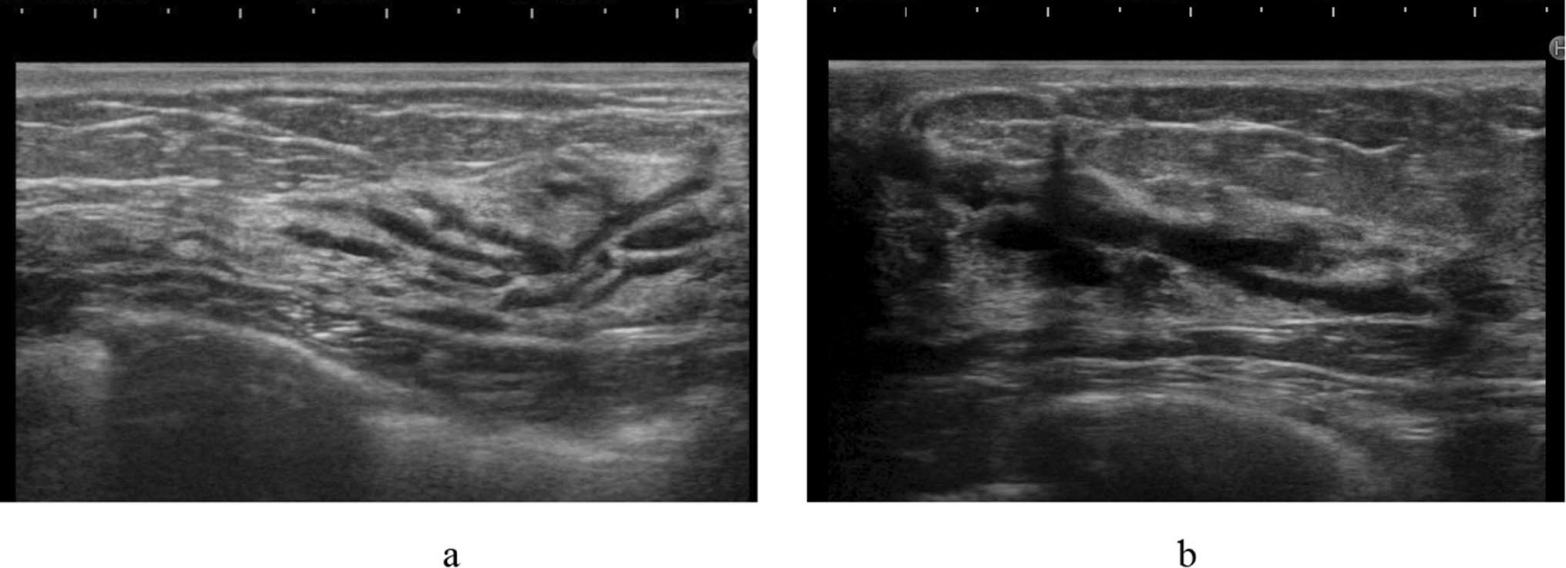
Typical images of abnormalities of the ducts

**Fig. 4:**
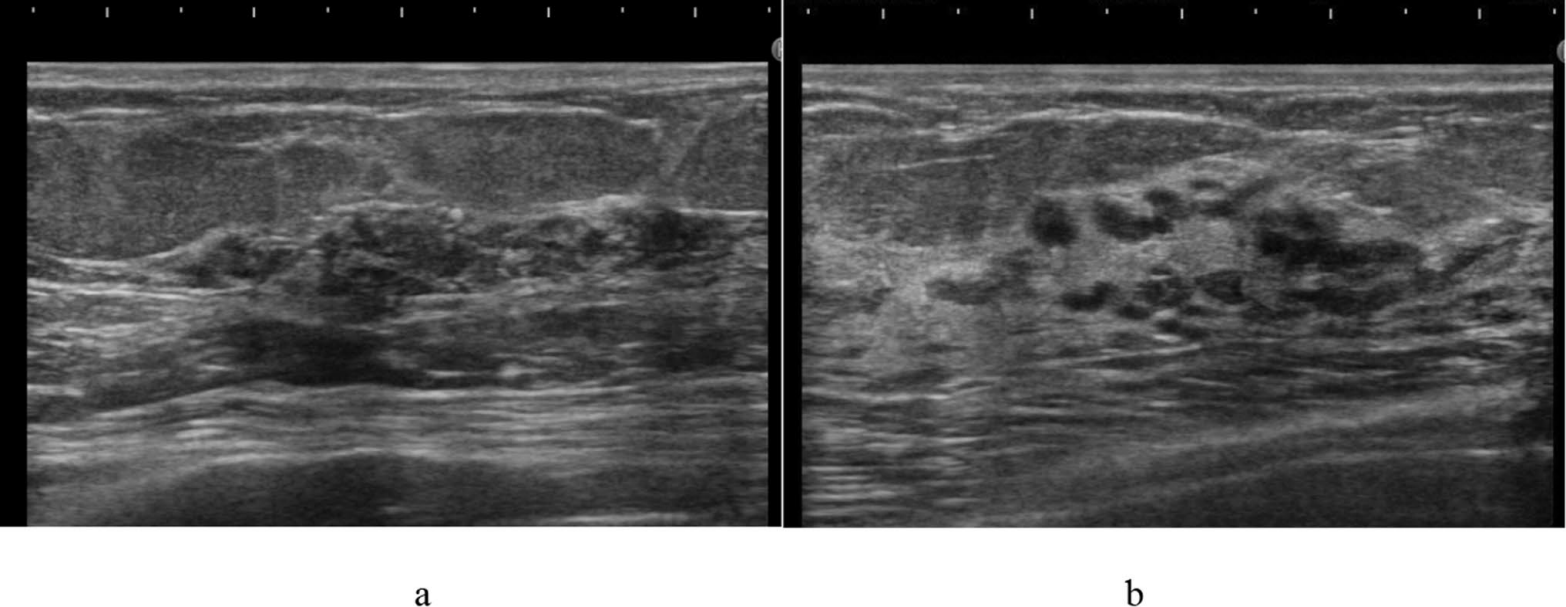
Typical images of hypoechoic areas in the mammary gland

**Fig. 5:**
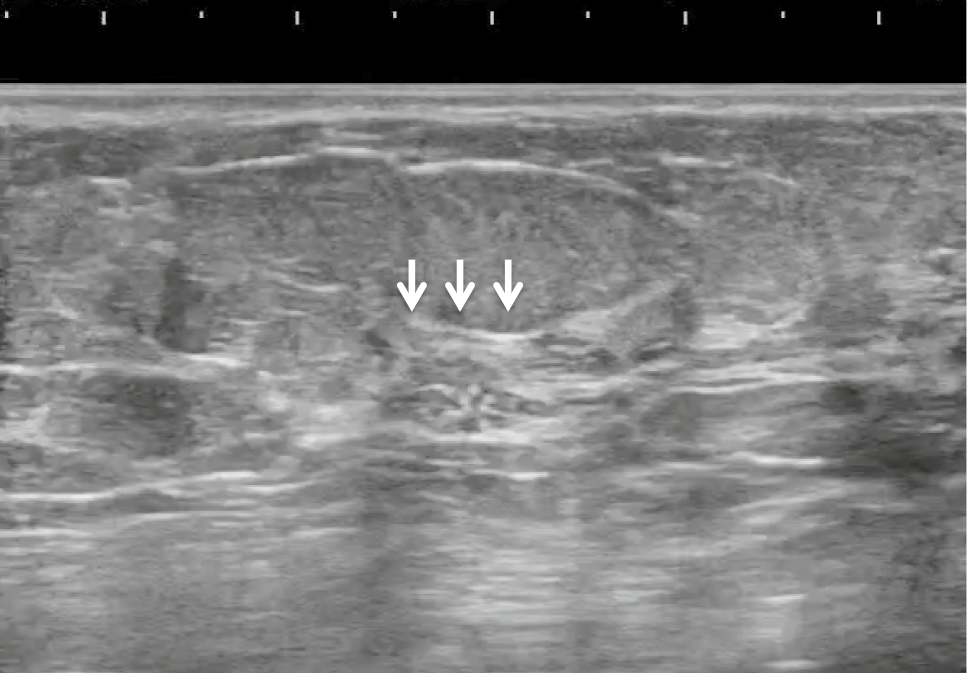
Typical image of echogenic foci without a hypoechoic area (arrows)

**Fig. 6:**
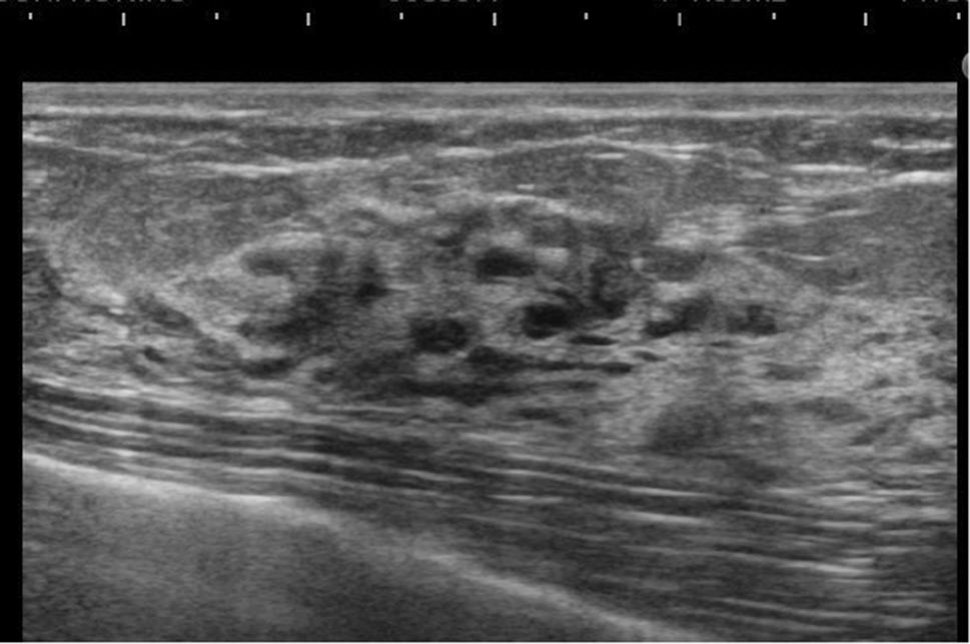
Typical image of clustered microcysts

**Fig. 7:**
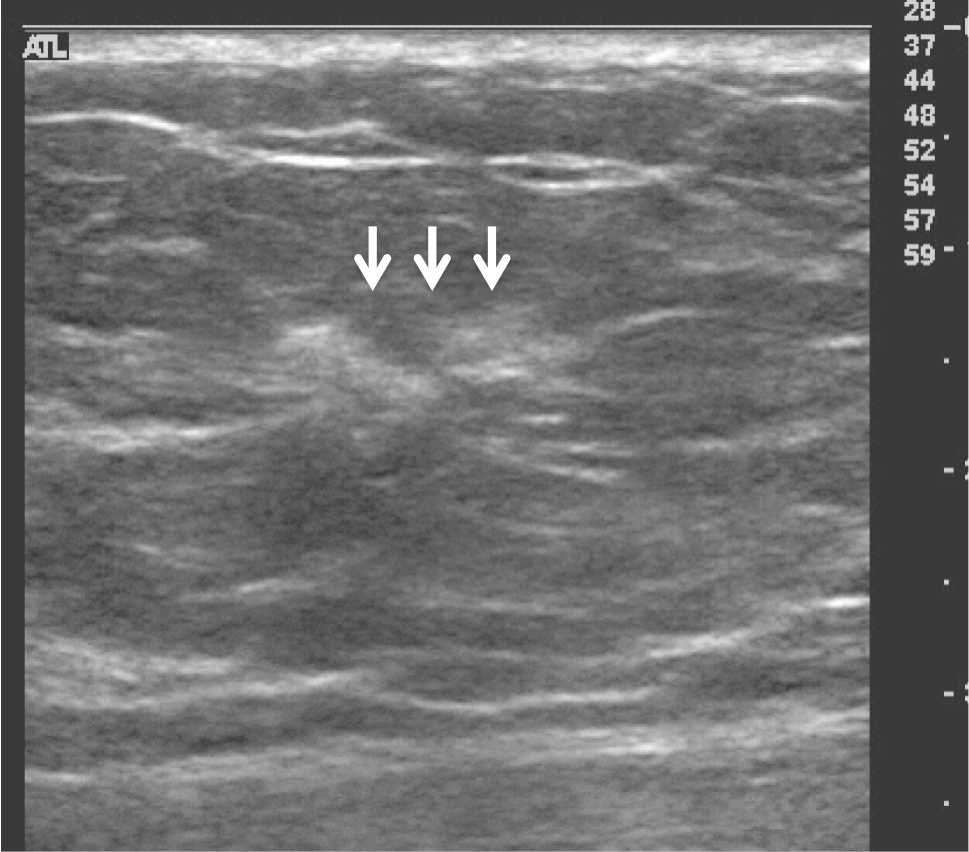
Typical image of architectural distortion (arrows)

The pathologic classification of breast cancer clearly distinguishes between invasive carcinoma and DCIS. However, it is difficult to clearly distinguish invasive cancer from DCIS on ultrasound images. Because DCIS is often recognized on ultrasound images as a lesion that does not form a mass, JABTS proposed the concept of non-mass abnormalities. Non-mass abnormalities are further classified as abnormalities of ducts, hypoechoic areas in the mammary gland, architectural distortion, clustered microcysts, and echogenic foci without a hypoechoic area. Representative images of each lesion are shown in Figs. 1, 2, 3, 4, 5, 6 and 7.

## The JABTS BC-02 study

DCIS is a lesion that presents a variety of ultrasound images, and the JABTS BC-02 study was conducted to clarify these variations. In this study, ultrasound images of 705 DCIS cases collected from 16 institutions in Japan were judged by a Centralized Image Interpretation Committee consisting of 14 breast ultrasound specialists. In the image classification, it was first determined whether the lesion was a mass or a non-mass abnormality. If the lesion was a mass, it was then classified as either solid or mixed. Non-mass abnormalities were subclassified into abnormalities of the ducts, hypoechoic areas in the mammary gland, architectural distortion, clustered microcysts, and echogenic foci without a hypoechoic area. When multiple image classifications were present on ultrasound images, the most predominant one was judged as the dominant type.

Finally, the frequency of each image classification was calculated. The results are shown in Table 1. Non-mass abnormalities and masses accounted for 60% and 40% of all lesions, respectively. Looking at each subclassification, hypoechoic areas in the mammary gland were the most common (50% of the total), followed by solid masses (31%), mixed masses (9%), and abnormalities of the ducts (8%). These four classifications accounted for 98% of the total. Echogenic foci without a hypoechoic area, architectural distortion, and clustered microcysts were very rare, accounting for about 1% of the total.

**Table 1:** Frequencies of subtypes among DCIS

Masses	277 (39.3%)
Solid masses	215 (30.5%)
Mixed masses	62 (8.8%)
Non-mass abnormalities	428 (60.7%)
Abnormalities of the ducts	57 (8.1%)
Hypoechoic areas in the mammary	50 (49.6%)
Architectural distortion	6 (0.9%)
Multiple small cysts (clustered microcysts)	3 (0.4%)
Echogenic foci without a hypoechoic area	12 (1.7%)
Total	705 (100%)

## Discussion

The ultrasound features of DCIS have been described in the literature. In general, the ultrasound findings of DCIS are classified into masses, ductal change (ductal abnormalities), calcification alone, and architectural distortion [3–6]). However, in recent reports, non-mass abnormalities (or lesions) have also been described as a finding in DCIS [7–9]).

The purpose of breast ultrasound is to diagnose benign or malignant disease. In addition, ultrasonography can be used to estimate the histologic type if the images are characteristic. For example, typical mucinous carcinoma is characterized by a well-defined, oval-shaped ultrasound image with high internal echoes [1]. The typical scirrhous carcinoma is irregularly shaped with a large depth/width ratio, a halo, and an attenuating posterior echo [1]. Thus, there is usually only one typical ultrasound image for each histologic type. However, DCIS is characterized by multiple typical lesion images. DCIS may be recognized as a mass or as a non-mass abnormality. DCIS that is recognized as a mass can be either a solid mass or a mixed mass. A mixed mass is very likely to be a DCIS. In the case of solid masses, relatively small, well-defined round masses are more likely to be DCIS. In non-mass abnormalities, hypoechoic areas and abnormalities of the ducts are typical images of DCIS. Segmental distribution of hypoechoic areas is highly suggestive of DCIS. Consecutive or multiple solid lesions within a single duct are also highly suggestive of DCIS. Understanding these typical ultrasound images may enable ultrasound diagnosis of DCIS.

## Conclusion

Ultrasound images of typical DCIS were described. The ultrasound images of DCIS were characterized by a wide range of variations from masses to non-mass abnormalities, with hypoechoic areas in the mammary gland being the most common, followed by solid masses. It was felt that a thorough understanding of typical DCIS images would make it possible to recognize the possibility of DCIS from ultrasound images.

## References

[1] Japan Association of Breast and Thyroid Sonology (JABTS) (2003). Guidelines for breast ultrasound: Management and diagnosis.

[2] Watanabe T, Yamaguchi T, Tsunoda H (2017). Ultrasound image classification of ductal carcinoma in situ (DCIS) of the breast: analysis of 705 DCIS lesions. Ultrasound Med Biol.

[3] Izumori A, Takebe K, Sato A (2010). Ultrasound findings and histological features of ductal carcinoma in situ detected by ultrasound examination alone. Breast Cancer.

[4] Park JS, Park YM, Kim EK (2010). Sonographic findings of high-grade and non-high-grade ductal carcinoma in situ of the breast. J Ultrasound Med.

[5] Wang LC, Sullivan M, Du H (2013). Appearance of ductal carcinoma in situ. Radiographics.

[6] Yang WT, Tse GMK (2004). Sonographic, mammographic, and histopathologic correlation of symptomatic ductal carcinoma in situ. AJR Am J Roentgenol.

[7] Jin ZQ, Lin MY, Hao WQ (2015). Diagnostic evaluation of ductal carcinoma in situ of the breast: ultrasonographic mammographic and histopathologic correlations. Ultrasound Med Biol.

[8] Lee MH, Ko EY, Han BK (2013). Sonographic findings of pure ductal carcinoma in situ. J Clin Ultrasound.

[9] Shin HJ, Kim HH, Kim SM (2008). Screening-detected and symptomatic ductal carcinoma in situ: differences in the sonographic and pathologic features. AJR.

